# Textile-Based Body Capacitive Sensing for Knee Angle Monitoring

**DOI:** 10.3390/s23249657

**Published:** 2023-12-06

**Authors:** Valeria Galli, Chakaveh Ahmadizadeh, Raffael Kunz, Carlo Menon

**Affiliations:** 1 Biomedical and Mobile Health Technology Laboratory, Department of Health Science and Technology, ETH Zurich, Balgrist Campus, Lengghalde 5, 8008 Zürich, Switzerland; chakaveh.ahmadizadeh@hest.ethz.ch (C.A.);; 2 Department of Mechanical and Process Engineering, ETH Zurich, 8092 Zürich, Switzerland

**Keywords:** motion capture, smart clothing, strain sensor, textile sensor, wearable technology

## Abstract

Monitoring human movement is highly relevant in mobile health applications. Textile-based wearable solutions have the potential for continuous and unobtrusive monitoring. The precise estimation of joint angles is important in applications such as the prevention of osteoarthritis or in the assessment of the progress of physical rehabilitation. We propose a textile-based wearable device for knee angle estimation through capacitive sensors placed in different locations above the knee and in contact with the skin. We exploited this modality to enhance the baseline value of the capacitive sensors, hence facilitating readout. Moreover, the sensors are fabricated with only one layer of conductive fabric, which facilitates the design and realization of the wearable device. We observed the capability of our system to predict knee sagittal angle in comparison to gold-standard optical motion capture during knee flexion from a seated position and squats: the results showed an R^2^ coefficient between 0.77 and 0.99, root mean squared errors between 4.15 and 12.19 degrees, and mean absolute errors between 3.28 and 10.34 degrees. Squat movements generally yielded more accurate predictions than knee flexion from a seated position. The combination of the data from multiple sensors resulted in R^2^ coefficient values of 0.88 or higher. This preliminary work demonstrates the feasibility of the presented system. Future work should include more participants to further assess the accuracy and repeatability in the presence of larger interpersonal variability.

## 1. Introduction

Monitoring human movement is relevant for health and fitness applications ranging from physical rehabilitation to athletic performance improvement. Current approaches are either spatially limited (optical motion capture, OMC) or expensive and obtrusive (suits equipped with inertial measurement units). Textile-based wearable technologies are valid alternatives thanks to their natural feel and unobtrusiveness. Several developments in the form of resistive, capacitive, and inductive textile sensors have been proposed in recent years [1,2] also thanks to the rapid development of fabrication techniques [3,4]. Capacitive sensors have a high linearity and a low drift/hysteresis as the response depends on the geometrical change of the electrodes [5], as follows:

(1) $$C=\frac{\varepsilon \;A}{d}$$

where *A* is the area of the electrodes, *d* the distance between the electrodes, and ε the permittivity of the dielectric material. When the electrodes are in direct contact with the body, the skin acts as a dielectric and the inner part of the body as one of the electrodes. Capacitive sensing has been used to track respiration rates based on the change in dielectric properties (ε) of the chest or abdomen with air intake [6,7], a reduction in skin thickness (*d*) on the abdomen during inhalation [8], a reduction in the distance between the electrodes (*d*) when breathing, or a change in the muscle contraction state [9]. Others have explored locomotion classification [10], swallowing, chewing, or speaking using similar approaches [9]. However, capacitive sensors using the body as an electrode have not been used for quantitative joint angle monitoring or precise tracking of other movements of the body. Previous works have demonstrated the use of soft and partly textile devices to monitor the knee angle using capacitive strain sensors [11,12,13], and we have previously reported a fully textile capacitive strain sensor tracking knee angles [14]. For these sensors, typical values of capacitance are in the picofarads range, due to the limited conductivity of conductive fabrics or sensor size. The effect of the parasitic capacitances of the circuit (e.g., connection lines to the electronic readout) is also usually in the order of 1–20 pF and can thus introduce an error that needs to be carefully accounted for.

We report a textile wearable device for knee angle monitoring through capacitive sensors in contact with the skin: by harnessing the conductivity of the human body, we shift the baseline capacitance of the sensor in the nanofarads range, allowing for an easier readout and a lower susceptibility to parasitic capacitances in the system. The device is easy to manufacture, comfortable to wear, and provides a venue for continuous movement monitoring through joint angle estimation. Moreover, the same working principle could be easily applied to other regions of the body for the detection of different movements. The sensor’s shape could also be optimized to accommodate different anatomical regions, while still employing the body as one of the electrodes.

## 2. Materials and Methods

### 2.1. Hardware

**Figure 1:**
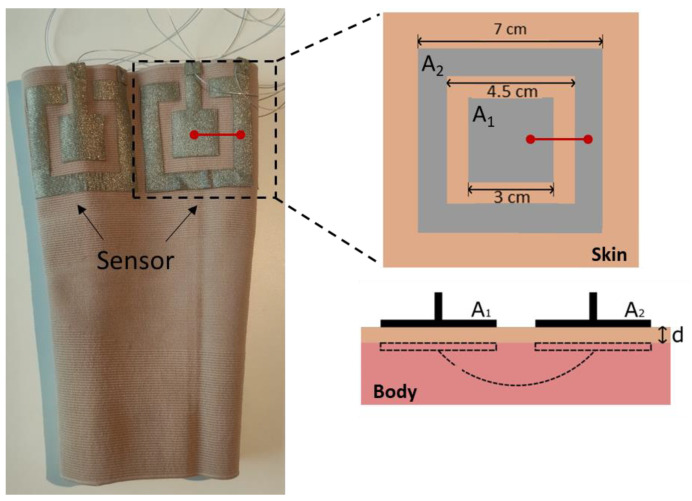
Knee sleeve and schematic of each sensor in frontal and side views with the corresponding dimensions. The lower right schematic shows how the body acts as a common electrode for both A1 and A2 electrodes.

The wearable device consisted of a commercial tight elastic knee sleeve with four textile capacitive sensors equally spaced in a circumferential direction. We refer to the sensors with the following names based on their position with respect to the anatomical direction: anterior (A), posterior (P), medial (M), and lateral (L). Each sensor was made of two concentric square electrodes (outer 7 cm, inner 3 cm, distance 0.75 cm, Figure 1). These dimensions were chosen as a trade-off between baseline capacitance (the bigger the electrodes, the higher and easier to detect) and size constraint on the knee sleeve (to fit four sensors in the circumferential direction). The electrodes were fabricated with commercial knitted conductive fabric with R~0.6 Ω/sq (Adafruit, New York, NY, USA) [15], which was laser-cut (Rayjet 50, Marchtrenk Austria) and secured to the sleeve using a hot melt adhesive web (Thermoweb, Wheeling, IL, USA) by ironing at 150° for 30 s (Cricut EasyPress 3, South Jordan, UT, USA). A Teflon-coated stainless steel conductive yarn (VN 12.1.2.175S, Bekaert, Belgium) was used for the connections. The connections were about 1 m long to allow for free movement while measuring capacitance. However, the employed yarn is highly conductive, with a linear resistance of about 30 Ω/m; thus, a negligible resistance was introduced to the system. The insulation helped minimize any noise introduced by the wires moving or touching each other or the skin during movement.

The electrodes were worn in contact with the skin, and the choice of their position above the kneecap was justified with a previous work that showed that the maximum skin stretch occurs in this anatomical location during knee flexion [16]. We did not put the sensors directly on the kneecap to avoid interference from other factors—such as the pressure of the kneecap on the electrodes—that would potentially introduce noise. The choice of coaxial structure was inspired by a previous work [17] and allowed for a compact sensor configuration, as compared to, e.g., two squares beside each other.

The two concentric electrodes of each capacitive sensor did not come in direct contact with each other during the experiments due to movement or stretching. In order to exploit two additional sensor configurations, we connected the two electrodes (red line in Figure 1) of the anterior and posterior sensors to form an anterior–posterior (AP) configuration and, similarly, the two electrodes of the medial and lateral sensors to form a medial–lateral (ML) configuration.

Impedance characteristics (capacitance, resistance, impedance phase, and magnitude) were measured with a precision inductance capacitance resistance (LCR) meter (Hioki IM3536, Nagano, Japan), with about 1% error as per the manufacturer’s specifications [18]. A trigger from the OMC system to the LCR ensured synchronization between the two devices during the measurements.

### 2.2. Experimental Protocol

All the tests were performed on a single participant. The following six different sensor configurations were used: single sensors (A, P, M, L) and paired sensors (A–P and M–L). For the paired sensors, the plates of the coaxial sensor were shorted so that the capacitance was measured between, i.e., the anterior and posterior sensors to form the antero-posterior (AP) or medio-lateral (ML) configurations. In such configurations, two sensors are connected in series, as the body acts as a common electrode for the two.

**Figure 2:**
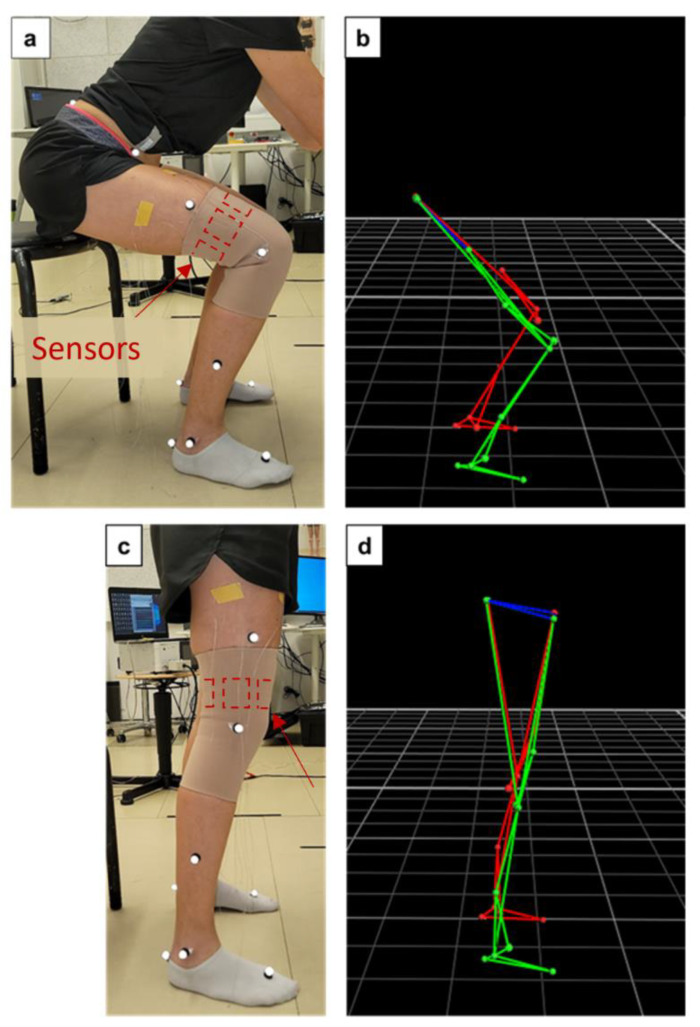
OMC tests with marker positioning and corresponding reconstruction from the data processing in squatting (**a**,**b**) and standing (**c**,**d**) positions. The position of the sensors on the inner side of the knee sleeve is indicated with the red dashed lines (**a**,**c**).

The ground truth for the sagittal knee angle was obtained using an OMC system (Vicon, 26 cameras). Reflective markers were placed on bony landmarks according to the Plug-in Gait model [19] on both legs to monitor lower body movements (Figure 2a,c). The following two movements were performed: flexing and extending the knee from a seated position, spanning about 90°, and squatting with a chair as the reference for the end position of the squat. All the activities were repeated 10 times (e.g., 10 squats per test).

### 2.3. Data Analysis

The raw data from the OMC were processed using the Vicon Nexus 2.12 software; Woltring filtering was applied on the trajectories before the processing of the Plug-in Gait model. We only considered the sagittal knee angle as flexion/extension are predominant in knee motions. The capacitance data were filtered with the Butterworth low pass filter (order 2, cut-off 1 Hz) to remove high frequency fluctuations of the capacitance not relevant to joint angle changes. Different features were extracted over a 100-milliseconds sliding window, and their trend was visually compared to the output sagittal angle: the 100 ms window was chosen after comparing the features obtained after 20, 50, 100, and 200 ms. Besides the filtered capacitance data, the following four features were retained: minimum, maximum, mean, and first derivative.

Several regression algorithms were tested following this pipeline: train-validation-test split (80%-10%-10%), hyperparameter tuning with 5-fold cross-validation on train/validation data, and prediction on unseen test data. The coefficient of determination R^2^, the root mean squared error (RMSE), and the mean absolute error (MAE) were used as the regression metrics. The analysis was performed in Python 3.7. Built-in algorithms with relatively low computational loads and model complexities were tested: Lasso, Ridge, Support Vector Machine, Stochastic Gradient Descent, XGBoost, Decision Tree, and random forest. The best-performing algorithm was chosen based on the highest R^2^ coefficient.

We also investigated the combination of the four single sensors and of the two “shorted” sensors (AP, ML) by concatenating their signals for each specific knee angle as follows. One of the test conditions (e.g., squat—anterior sensor) was taken as a reference; for each data point, i.e., for each sagittal angle, all the features (raw capacitance data, minimum, maximum, mean, 1st derivative of capacitance) corresponding to that specific angle within a tolerance of 0.5 degrees were concatenated. For example, for the combination of the four sensors, the feature dataset had twenty columns (features): five columns per test times four sensor locations. Table 1 shows an example of such a feature set.

Similarly, the combination of the data for the “shorted” configurations (A–P and M–L) yielded a feature dataset of 30 columns (features).

## 3. Results

**Figure 3:**
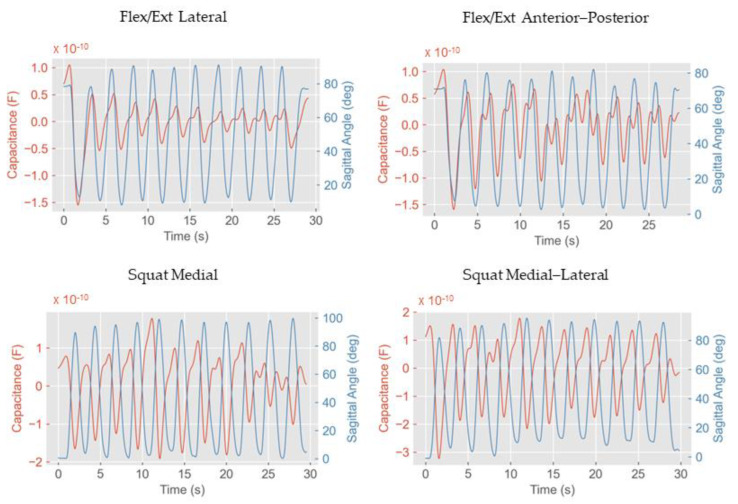
Capacitance vs. sagittal angle: examples from different locations and tests. For reference, we have reported the worst- and best-performing sensors (flex/ext lateral and squat medial–lateral, respectively).

Representative graphs of the capacitance from the sensors and sagittal knee angle from the OMC are depicted in Figure 3**.** The random forest regression resulted in the best prediction capability among all the algorithms. The results from all the other algorithms are shown in the Supplementary Materials (Table S1). The posterior sensor yielded the least accurate predictions in both the flexion/extension and squat tests. Generally, the squat tests yielded better results than the flexion/extension from a seated position. For the squat tests, relatively accurate angle predictions were obtained with all the sensor configurations with R^2^ of 0.89 or above (Table 1).

**Figure 4:**
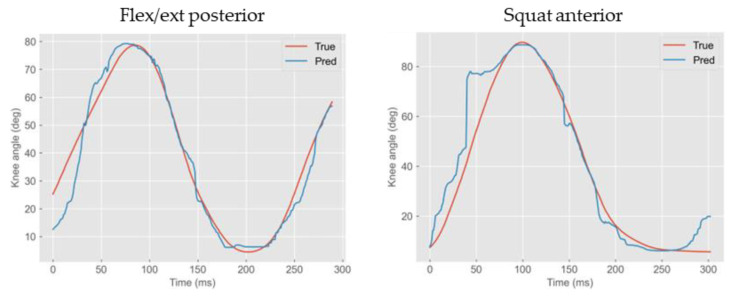
Sample results of angle predictions for the test set in different sensor locations and movements.

For flexion/extension, only the medial and lateral sensors yielded R^2^ values below 0.80 (Table 2). For a visual comparison, the predicted angle vs. the ground truth for four different tests with different regression performances are shown in Figure 4: the flexion/extension from the seated position (top two graphs) gave worse predictions than the squats (bottom graphs).

The combination of the four single sensors and the two “shorted” sensors resulted in R^2^ > 0.88, RMSE < 8°, and MAE < 6° for all the movements (Table 3). The regression results on the combined sensors for the other regression algorithms are shown in the Supplementary Materials (Table S2).

## 4. Discussion

We have developed a simple-to-manufacture and comfortable-to-wear textile sensing system for knee motion tracking. We have exploited the natural conductivity of the human body to use it as one of two electrodes of textile capacitive sensors. In this configuration, the skin acts as a dielectric layer.

When the knee angle changes, the shape of the sensors is affected in the following two ways: (1) the distance between the electrodes of the parallel plate capacitor decreases as the skin stretches and becomes thinner; (2) the area of the stretchable conductive electrodes increases or decreases based on the position of the sensor. For example, the anterior sensor undergoes stretching when the knee is flexed.

In this specific modality with electrodes in contact with the skin, multiple factors contribute to the changes in capacitance of the sensors, including the following: (a) movements/stretching of the electrodes as muscles contract and relax, and (b) changes in the composition of the skin and the underlying tissues underneath the textile electrodes.

Evidence from the literature is available to support both factors. This modality has mostly been used to monitor respiration rates by placing the electrodes on either side of the chest/abdomen: it is as assumed that the addition and reduction in air in the lungs during inhalation and exhalation causes a change in the permittivity of the dielectric (ε ≈ 1 for air, ε ≈ 80 for water), which, in turns, leads to a capacitance change in the sensors [6,7,8]. The measured capacitance reported in some of these studies ranges between approximately 120 pF and 550 pF, depending on the configuration of the electrodes [6]. Terazawa et al. tested this modality for respiration rate monitoring and placed the electrode on the abdomen. They concluded that changes in the thickness of the skin (by inhaling, the skin thins) result in a capacitance change [8]. These studies provide evidence for factor (b) as the underlying cause of capacitance changes.

Cheng et al. reported on sensors mounted in a similar fashion to the ones in this work but without contact with the skin, as their sensors were worn on top of pants [9]. They concluded that the changes in capacitance are mainly caused by movements of the electrodes, i.e., factor (a). The range of capacitance is not reported as their output is converted to voltage and amplified in three stages. Similar conclusions on the variation of capacitance based on the leg shape and inner tissues (muscle) were reported in a study with an array of capacitive sensors on the thigh in direct contact with the skin [10].

To understand which one of these factors is dominant, we performed tests with the same prototype worn inside-out (with the capacitive sensors not in contact with the leg): in this configuration, only factor (a) contributes to the response of the sensors. We observed changes in capacitance in the range of 5 pF, about 20-folds-lower than the changes observed when the electrodes were in contact with the skin (approximately 100 pF). As we were able to record meaningful signals both without (factor (a) only) and with contact with the skin (i.e., factor (a) and (b)), but with different amplitudes, we conclude that both the aforementioned factors concur in the changes in the signal.

However, it should be noted that the cause of these factors is not in the same direction and that some cancel each other out. For example, when the muscle underneath the electrode is contracted, the thickness of the skin reduces, resulting in a higher capacitance. However, with the same muscle contraction, electrodes become farther apart, resulting in a decrease in capacitance. This was evident in the tests we performed with skin contact, where we observed an increase in the capacitance of the sensor placed on the anterior side during knee flexion, while, in the tests without skin contact, we observed the opposite. Since in our tests with OMC that combine both these factors we observe a decrease in capacitance in the same location during knee flexion, we conclude that the effect of the movement of electrodes combined with skin contact is dominant.

We observed the opposite direction of changes in the anterior and lateral directions during knee flexion and extension (Figure 3, left panes). This was expected as the antagonist muscles work in an opposite manner during knee flexion, i.e., the muscles on the anterior side are relaxed during flexion while the muscles on the posterior side are contracted. As the medial and lateral muscles are located in a way in which they can be affected by both muscle groups, based on our results, we conclude that the effect of posterior muscles was more prominent than that of the anterior ones.

From Figure 3, it can be noted that the capacitance is not fully aligned with the sagittal knee angle: this is to be expected based on our hypothesis of the two factors causing capacitance changes upon knee flexion. Additionally, the magnitude of the signal from the capacitive sensors is not fully consistent throughout the movement repetitions. We hypothesize that this may be due to slight movements of the electrodes with respect to the skin. Although such movements could be prevented using adhesive patches on the skin instead of textile electrodes on the knee sleeve, the use of adhesives would reduce the level of comfort of the wearable system. Nonetheless, we could achieve an acceptable accuracy in knee angle prediction even for the worst-performing sensor with regression algorithms (R^2^ = 0.76) without sacrificing the comfort of our wearable device.

There are some advantages of such a modality (contact with the body) over other textile-based sensing modalities. First, the baseline capacitance of the sensor is higher compared to standard textile capacitors thanks to the very thin dielectric layer (skin) and the conductance of the body, which facilitates readout with electronic circuits. Also, artifacts from a slippage between sensorized clothing and the skin are prevented, as the textile electrodes sit tightly in contact with the leg and are pressed against it by the knee sleeve.

Although we could not investigate the sensitivity of the sensor with controlled bench tests—given that the participant’s body is part of the sensing system itself—we have compared the ability of this method for the prediction of knee angle to similar works in the literature. Our results are in line with similar reports of textile-based capacitive sensors that, however, employ custom fabrication of the electrodes (RMSE~2°) [11] or multiple sensors and encapsulation of the electrodes in Ecoflex (RMSE~4°) [12]. A similar performance was also obtained with resistive textile sensors (R2 = 0.97 [20], with an RMSE~4° [21]), although resistive sensors are known to be more prone to drift and hysteresis [5].

When comparing the angle prediction capability between the two movements, the squats gave a better performance compared to the knee flexion/extension from a seating position. The underlying factors driving the sensors’ response were technically the same for the two movements, so it is difficult to draw conclusions based on the different performance in the two movements. We hypothesize that the reason may be that the squat movement was more controlled and easier to repeat consistently; therefore, knee sagittal angle was more consistent between the repetitions and so were the leg movement and muscular effort in the monitored area. The slow knee flexion/extension from a seating position required a higher muscular effort to control the movement, and the repeatability of the sagittal angle across the tests was lower. As expected, the posterior sensor had the worst prediction capability: this could be due to wrinkling of the electrodes and/or imperceptible change in skin thickness during flexion. The combination of anterior–posterior and medial–lateral sensors gave good results; however, it did not enhance the regression performance compared to single sensors. For the squat tests, all the sensors yielded very good results, showing the potential of the wearable device for knee angle estimation with capacitive sensors. As expected, the combination of all the sensors improved the results compared to single sensors, with R^2^ > 0.98 for most movements.

With regards to the design of the system and the number of sensors, we have chosen to start with a simple configuration with only four electrodes to explore the feasibility of this method. We have maximized the electrodes’ area to amplify the baseline capacitance of the sensors. The addition of multiple smaller sensors would increase the complexity but also possibly give information on the knee angle in the other two planes (frontal and transverse). Furthermore, a more advanced system with more sensors would allow researchers to improve accuracy for angle prediction in less controlled movements (e.g., flexion/extension where the knee angle was not as consistent between the repetitions as it was for the squats).

With specific regard to the prediction of knee angle in 3D, higher-density sensor grids have been previously used for this scope, together with machine learning algorithms [22]. Acquiring data from a larger number of sensors and participants would allow researchers to select the optimal sensor location for joint angle prediction, as shown in similar works in the field [22,23]. Moreover, further movements that highlight frontal and transverse knee angles—e.g., single leg squats, step down, sidestep cut, or others—could be employed to expand the system to 3D angle monitoring.

The selection of sensor locations should be further investigated in more complex movements, e.g., with the aim of predicting frontal and transverse angles. Future developments should also include the collection of data on multiple participants and with additional movements to ensure repeatability. More complex or computationally heavy algorithms were not explored as the standard regression models already yielded a high predictive capability. However, with the acquisition of more data, neural networks could be a good alternative to standard regression algorithms to enhance prediction accuracy.

## 5. Conclusions

The proposed textile-based wearable device for monitoring human movements showed promising results in terms of knee angle prediction accuracy and provides an easy-to-manufacture and comfortable-to-wear system for continuous movement monitoring. The accuracy of angle predictions in the sagittal plane reached low errors compared to the gold-standard measurement method showing the comparable performance of this device to other state-of-the-art wearable technologies, while providing higher ranges of capacitive signals. The expansion of testing to multiple participants and the selection of the most useful sensor locations are the subject of future research to further improve the system.

## Figures and Tables

**Table 1:** Structure of the feature dataset after the combination of different sensors (units = Farads).

Flex/Ext Anterior					…	Flex/Ext Posterior				
C	C_min_	C_max_	C_mean_	C’	…	C	C_min_	C_max_	C_mean_	C’
1.60 × 10^−11^	2.68 × 10^−13^	2.82 × 10^−11^	2.08 × 10^−11^	−1.34 × 10^−12^	…	1.41 × 10^−10^	2.24 × 10^−11^	1.41 × 10^−10^	9.68 × 10^−11^	−2.50 × 10^−12^
…	…	…	…	…	…	…	…	…	…	…
Col 1										Col 20

**Table 2:** Results for random forest regression on single sensors or sensor pairs. A: anterior, M: medial, L: lateral, P: posterior, AP: anterior–posterior, ML: medial–lateral.

Test	Sensor	R^2^	RMSE (deg)	MAE (deg)
Flex/Ext	A	0.83	10.79	6.93
	L	0.77	12.19	9.89
	M	0.76	13.31	10.34
	P	0.95	5.48	3.56
	A–P	0.82	11.15	5.99
	M–L	0.86	10.46	6.74
Squat	A	0.93	8.13	4.9
	L	0.93	8.28	6.09
	M	0.99	4.15	3.28
	P	0.95	7.59	6.58
	A–P	0.89	11.56	6.39
	M–L	0.94	8.19	6.51

**Table 3:** Results for random forest regression on a combination of single sensors or sensor pairs. A: anterior, M: medial, L: lateral, P: posterior, AP: anterior–posterior, ML: medial–lateral.

Test	Sensor Combination	R^2^	RMSE (deg)	MAE (deg)
Flex/Ext	A + L + M + P	0.88	7.9	2.62
	AP + ML	0.97	4.03	3.31
Squat	A + L + M + P	0.99	2.61	2.02
	AP + ML	0.94	7.52	5.7

## Data Availability

The raw data are available on the ETH research data collection with Available online: https://www.research-collection.ethz.ch/handle/20.500.11850/638914 (accessed on 26 October 2023).

## Supplementary Materials

The following supporting information can be downloaded at: https://www.mdpi.com/article/10.3390/s23249657/s1. Figure S1: Schematic representation of the knee angles in the three anatomical planes. Table S1: Regression metrics for all algorithms on single sensors tests. Table S2: Regression metrics for all algorithms on combined sensors tests.
