# Supplementary material for: Textile-Based Body Capacitive Sensing for Knee Angle Monitoring
Source: Sensors (Basel). 2023 Dec 6;23(24):9657. doi: 10.3390/s23249657 (PMC10747625; doi:10.3390/s23249657)
Supplement: Supplementary file 1 [file sensors-23-09657-s001.zip › sensors-2713477-supplementary.pdf]

Supplementary Materials

Definition of knee angles in the three anatomical planes.

For reference, we report below the definition of the knee angles in the three planes:

- knee flexion: angle between the long axis of the shank and the long axis of the thigh in the sagittal plane;
- knee ab/adduction: angle between the long axis of the thigh and the long axis of the shank in the frontal plane;
- knee rotation: angle between the long axis of the shank and the long axis of the thigh as observed in the transverse plane.

A more detailed description of the angles calculation from the optical motion capture system's manufacturer is available from the documentation (https://docs.vicon.com/display/Nexus212/Plug-in+Gait+kinematic+variables).

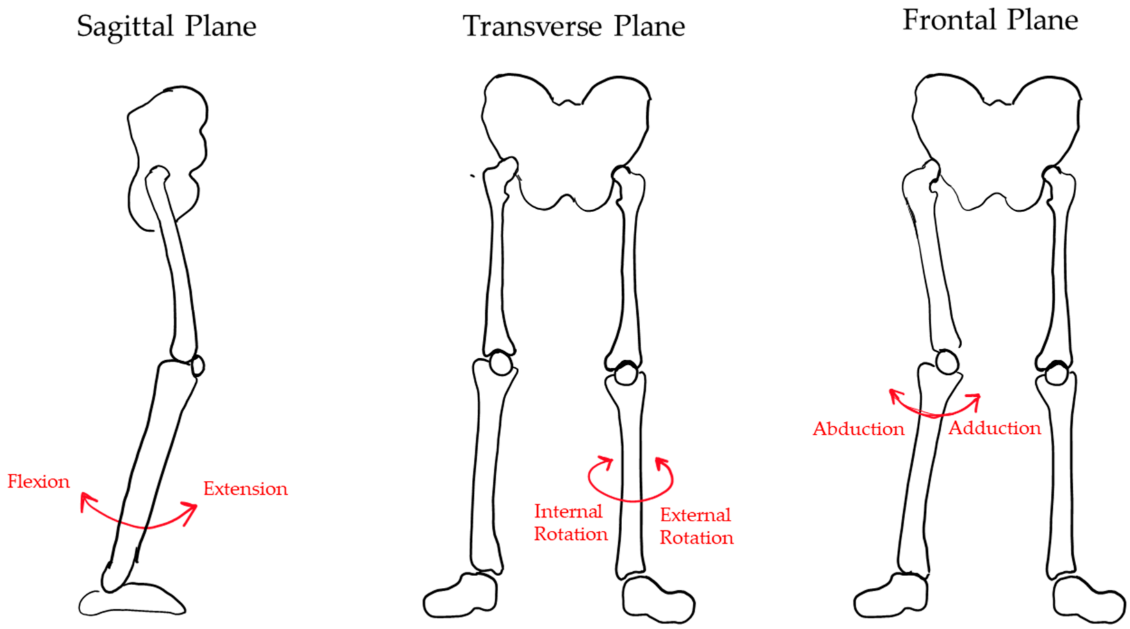

Figure S1: Schematic representation of the knee angles in the three anatomical planes.

Table S1: Regression metrics for all algorithms on single sensors tests.

Model	Test	Test	R2	RMSE	MAE
Decision Tree Regression	Flex/Ext	A	0.83	10.9	7.16
		AP	0.67	15.17	7.9
		L	0.26	21.83	15.6
		M	0.74	13.85	10.38
		ML	0.85	10.8	6.72
		P	0.93	6.67	4.68
	Squat	A	0.91	9.15	6.01
		AP	0.85	13.13	7.3
		L	0.88	10.68	7.06

		M	0.97	5.6	4.27
		ML	0.94	7.81	4.72
		P	0.95	7.28	6.3
Lasso Regression	Flex/Ext	A	0.94	6.36	5.58
		AP	0.9	8.44	6.86
		L	0.83	10.38	8.62
		M	0.86	10.21	9.12
		ML	0.96	5.47	4.6
		P	0.97	4.23	3.17
	Squat	A	0.97	4.85	3.59
		AP	0.97	6.1	5.01
		L	0.94	7.23	5.46
		M	0.98	5.06	4.04
		ML	0.93	8.41	6.71
		P	0.89	11.16	8.99
Linear Regression	Flex/Ext	A	0.94	6.46	5.68
		AP	0.9	8.46	6.88
		L	0.83	10.58	8.81
		M	0.86	10.2	9.11
		ML	0.96	5.48	4.59
		P	0.97	4.3	3.22
	Squat	A	0.98	4.7	3.42
		AP	0.97	6.12	5.02
		L	0.96	6.39	4.94
		M	0.98	5.03	4.03
		ML	0.93	8.46	6.73
		P	0.98	4.91	3.82
**Random Forest Regression**	**Flex/Ext**	**A**	**0.83**	**10.79**	**6.93**
		**AP**	**0.82**	**11.15**	**5.99**
		**L**	**0.77**	**12.19**	**9.89**
		**M**	**0.76**	**13.31**	**10.34**
		**ML**	**0.86**	**10.46**	**6.74**
		**P**	**0.95**	**5.48**	**3.56**
	**Squat**	**A**	**0.93**	**8.13**	**4.9**
		**AP**	**0.89**	**11.56**	**6.39**
		**L**	**0.93**	**8.28**	**6.09**
		**M**	**0.99**	**4.15**	**3.28**
		**ML**	**0.94**	**8.19**	**6.51**
		**P**	**0.95**	**7.59**	**6.58**
Ridge Regression	Flex/Ext	A	0.94	6.26	5.47
		AP	0.9	8.31	6.7
		L	0.86	9.63	7.9
		M	0.86	10.22	9.13
		ML	0.96	5.52	4.66

		P	0.97	4.07	3.03
	Squat	A	0.98	4.73	3.46
		AP	0.97	6.04	4.92
		L	0.95	6.52	4.96
		M	0.98	5.16	4.1
		ML	0.93	8.43	6.72
		P	0.89	11.14	8.92
Stochastic Gradient Descent Regression	Flex/Ext	A	0.95	5.89	5.09
		AP	0.9	8.26	6.64
		L	0.95	5.67	4.54
		M	0.86	10.25	9.16
		ML	0.94	6.59	5.21
		P	0.98	3.61	2.9
	Squat	A	0.98	4.81	3.52
		AP	0.97	6.08	4.9
		L	0.95	7.01	5.27
		M	0.98	5.21	4.15
		ML	0.94	8.13	6.64
		P	0.97	5.89	3.96
Support Vector Regression	Flex/Ext	A	0.98	3.44	2.85
		AP	0.98	3.86	3.08
		L	0.73	13.13	10.26
		M	0.92	7.53	6.74
		ML	0.93	7.07	5.35
		P	0.96	5	3.58
	Squat	A	0.99	2.39	1.65
		AP	0.99	2.66	2.31
		L	0.94	7.41	4.92
		M	0.99	3.8	3.1
		ML	0.99	2.6	2.02
		P	0.89	11.18	8.69
XGBoost Regression	Flex/Ext	A	0.74	13.47	11.5
		AP	0.71	14.3	10.4
		L	0.53	17.4	13.36
		M	0.75	13.6	11.14
		ML	0.69	15.44	10.81
		P	0.67	14.05	12.52
	Squat	A	0.9	9.63	6.43
		AP	0.6	21.72	16.84
		L	0.86	11.27	9.79
		M	0.83	14.21	11.13
		ML	0.7	18.18	14.47
		P	0.37	27.13	24.22

Table S2: Regression metrics for all algorithms on combined sensors tests.

Model	Test	Sensors	R2	RMSE	MAE
Decision Tree Regression	Flex/Ext	A + L + M + P	0.88	7.79	2.96
		AP + ML	0.89	7.93	4.71
	Squat	A + L + M + P	0.84	12.31	8.8
		AP + ML	0.96	5.78	3.34
Lasso Regression	Flex/Ext	A + L + M + P	0.97	3.87	3.32
		AP + ML	0.96	4.98	3.93
	Squat	A + L + M + P	1	1.57	1.08
		AP + ML	0.91	8.76	7.78
Linear Regression	Flex/Ext	A + L + M + P	0.97	3.9	2.81
		AP + ML	0.95	5.04	3.98
	Squat	A + L + M + P	1	1.44	1.03
		AP + ML	0.91	8.71	7.74
Random Forest Regression	Flex/Ext	A + L + M + P	0.88	7.9	2.62
		AP + ML	0.97	4.03	3.31
	Squat	A + L + M + P	0.99	2.61	2.02
		AP + ML	0.94	7.52	5.7
Ridge Regression	Flex/Ext	A + L + M + P	0.97	3.72	2.93
		AP + ML	0.95	5.15	4.07
	Squat	A + L + M + P	1	1.36	1.01
		AP + ML	0.91	8.69	7.81
Stochastic Gradient Descent	Flex/Ext	A + L + M + P	0.97	3.65	2.85
		AP + ML	0.95	5.23	4.07
	Squat	A + L + M + P	1	1.36	1.03
		AP + ML	0.92	8.48	7.73
Support Vector Regression	Flex/Ext	A + L + M + P	0.99	2.52	1.45
		AP + ML	0.98	3.65	2.77
	Squat	A + L + M + P	1	1.68	1.07
		AP + ML	0.98	3.85	3.08
XGBoost Regression	Flex/Ext	A + L + M + P	0.95	4.99	3.74
		AP + ML	0.98	2.97	2.32

	Squat	A + L + M + P	0.97	5.01	3.59
		AP + ML	0.79	13.6	9.5
